# Postoperative Morbidity Is Not Associated with a Worse Mid-Term Quality of Life After Colorectal Surgery for Colorectal Carcinoma

**DOI:** 10.3390/jcm14145167

**Published:** 2025-07-21

**Authors:** Maximilian Brunner, Theresa Jendrusch, Henriette Golcher, Klaus Weber, Axel Denz, Georg F. Weber, Robert Grützmann, Christian Krautz

**Affiliations:** Department of General and Visceral Surgery, Friedrich-Alexander-University Erlangen-Nürnberg (FAU), Krankenhausstraße 12, 91054 Erlangen, Germany; theresa.jendrusch@freenet.de (T.J.); henriette.golcher@uk-erlangen.de (H.G.); klaus.weber@uk-erlangen.de (K.W.); axel.denz@uk-erlangen.de (A.D.); georg.weber@uk-erlangen.de (G.F.W.); robert.gruetzmann@uk-erlangen.de (R.G.); christian.krautz@uk-erlangen.de (C.K.)

**Keywords:** colorectal carcinoma, colorectal surgery, quality of life, patient-related outcome, morbidity

## Abstract

**Objectives**: The aim of the present study was to investigate the impact of postoperative morbidity on mid-term quality of life and patient-related outcome (PRO) parameters after colorectal surgery for colorectal carcinoma. **Methods**: Quality of life and perioperative data were prospectively collected from 99 adult patients treated for colorectal carcinoma—56 patients with colonic carcinoma and 43 with rectal carcinoma, all of whom underwent R0 colorectal resection, at the University Hospital Erlangen between 2018 and 2021. Quality of life data (EQL C29 and C30) were assessed before the start of treatment and one year after. Patients were grouped based on the presence or absence of postoperative morbidity, and their quality of life was compared between the two groups. **Results**: In the colonic carcinoma cohort, global quality of life and emotional functioning showed significant improvement from pre-treatment to the one-year follow-up (63 vs. 72, *p* = 0.012 and 63 vs. 76, *p* = 0.009, respectively). Among the symptom scales, five items improved, while two worsened. Patients who experienced postoperative morbidity (32% in the colonic carcinoma group) did not exhibit worse outcomes in functioning or symptom scales compared to those without morbidity (4 items improved and 1 worsened in the morbidity group vs. 3 improved and 1 worsened in the no-morbidity group). The rectal carcinoma cohort demonstrated a decline in quality of life from pre-treatment to the one-year follow-up. Two functioning scales worsened significantly (physical function: 89 vs. 83, *p* < 0.001; role function: 81 vs. 68, *p* = 0.009), and twelve symptom scales showed deterioration, with only two symptom scales improving. Postoperative morbidity (33% in the rectal carcinoma group) did not result in more pronounced impairments compared to those without morbidity. The morbidity group experienced 2 worsened and 0 improved items, while the no-morbidity group had 10 worsened and 1 improved item. **Conclusions**: Postoperative morbidity was not significantly associated with a worse quality of life at one-year follow-up after treatment of colorectal carcinomas, including colorectal resections, compared to patients who did not develop postoperative morbidity.

## 1. Introduction

Colorectal carcinoma represents the third most common cancer worldwide, with approximately 2 million new diagnoses and 1 million deaths annually, making it the second leading cause of cancer-related deaths each year [1]. Improved surgical techniques, multimodal treatment concepts and advances in metastasis treatment have led to continuously improving survival rates in the last decades [2].

Beyond survival, maintaining a good quality of life is another crucial aim of treatment [3]. As survival rates improve, more patients live with the potential side effects of treatment. Thus, measuring quality of life and understanding factors influencing quality of life and patient-related outcomes are essential to enhance the well-being of patients with colorectal carcinoma.

Most studies indicate that quality of life remains unchanged between preoperative assessment and 12–18 months follow-up, with some even reporting improvements and some worsening [4,5,6,7,8,9]. However, impairments in bowel function are frequently observed after colorectal resections and additionally affections of urinary and sexual function after treatment of rectal cancer [6,10,11,12]. These functional impairments can significantly influence well-being and quality of life [6,10,13]. Additionally, studies have identified further factors that impact quality of life: disease recurrence is a known negative factor, and the need for a stoma is frequently discussed as an influential factor [14,15,16]. Moreover, a current analysis of over 3000 patients from Germany identified that older age, male gender, better education, and private insurance were associated with improved patient-related outcomes [7].

Postoperative complications after colorectal surgery, a cornerstone in the therapy for colorectal carcinoma, relevantly impact well-being during the postoperative hospital stay and in the short term [17,18]. However, their influence on mid- and long-term quality of life has not been extensively investigated [19,20,21]. In a prospective cohort study, Bosma et al. evaluated the influence of postoperative complications, classified by the Clavien–Dindo system, on quality of life after colorectal surgery. They found that only major complications (Clavien–Dindo grade III–V) were significantly associated with a temporary decline in physical and psychological quality of life at six weeks postoperative, while minor complications (grade I–II) had no significant effect compared to patients without complications. Importantly, quality of life returned to baseline levels in all groups by 12 months, suggesting that the negative impact of complications on quality of life is largely confined to the early postoperative period [19]. In a related study, Bosma et al. also examined health status, anxiety, and depressive symptoms following complicated and uncomplicated colorectal surgeries. Patients with complicated postoperative courses reported significantly worse health status and higher anxiety and depression levels shortly after surgery. However, these differences diminished over time, and by 12 months postoperatively, psychological symptoms and overall health status had improved to levels comparable to those without complications [20]. This underscores the importance of early psychological support for patients experiencing complications. Extending these findings, Orive et al. conducted a 5-year follow-up study assessing anxiety, depression, health-related quality of life (HRQoL) and mortality in colorectal cancer patients. They found that higher levels of anxiety and depression were significantly associated with poorer HRQoL and increased mortality risk over the long term. The study highlights that psychological distress remains a critical factor affecting both survival and quality of life years after colorectal cancer treatment, emphasizing the need for ongoing psychological assessment and support as a core component of survivorship care [21].

Given the prevalence of complications in colorectal surgery and their potential to trigger further interventions, readmissions, or psychological distress, a deeper understanding of their lasting impact is crucial. Moreover, it remains unclear whether the impact of postoperative morbidity differs between patients with colon and rectal carcinoma. Given the distinct surgical as well as therapy approaches (e.g., neoadjuvant chemoradiation), complication profiles, and functional outcomes associated with these tumor locations, a differentiated analysis is essential to tailor supportive care and postoperative follow-up strategies more effectively.

Therefore, the primary objective of this study was to investigate whether postoperative morbidity has a negative impact on mid-term quality of life in patients undergoing colorectal resections for colorectal malignancies.

## 2. Materials and Methods

**Figure 1:**
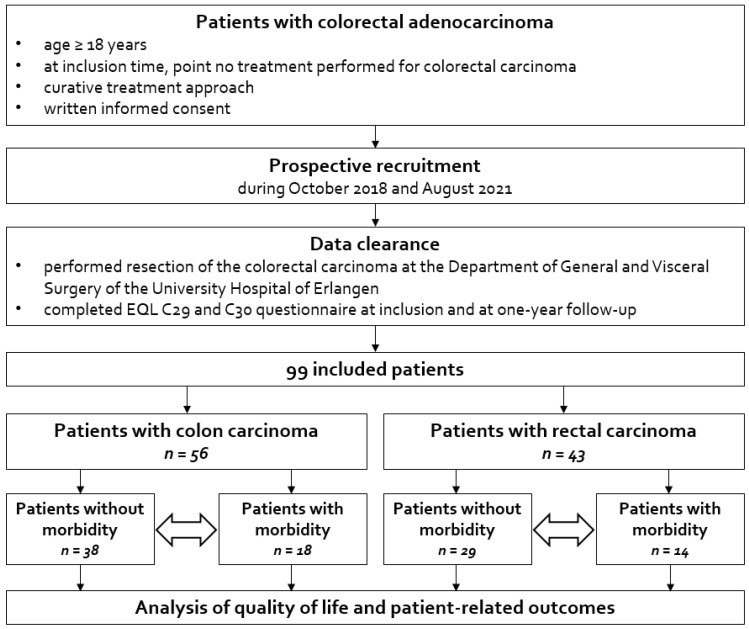
Flow chart of the study.

This was a single-center observational study with prospectively collected QoL data at baseline and one-year follow-up, analyzed retrospectively. The study included all patients with following inclusion criteria: (1) age ≥ 18 years; (2) diagnosis of colorectal carcinoma; (3) at inclusion time point no treatment performed for colorectal carcinoma; (4) curative treatment approach, including performed resection of the colorectal carcinoma at the Department of General and Visceral Surgery of the University Hospital of Erlangen; (5) written informed consent; and (6) completed EQL C29 and C30 questionnaire at inclusion and at one-year follow-up. Patients were excluded if they met any of the following criteria: (1) age younger than 18 years; or (2) missing EQL C29 and C30 questionnaire at any timepoint. Recruitment were performed prospectively during October 2018 and August 2021 (Figure 1).

Assessment of quality of life and patient-related outcomes (PROs) were performed prospectively using the well validated EQL C29 and C30 questionnaires [22]. All patients answered both questionnaires at the timepoint of inclusion and at a one-year follow-up (±30 days). Additionally, data about the included patients were collected including patient demographics, tumor characteristics, therapeutic details, histopathological results and postoperative outcomes. Histopathological classification of all carcinomas occurred in accordance with the 8th edition of the UICC TNM classification [23]. Morbidity was defined as any deviation from the normal postoperative course and classified according to the Clavien–Dindo classification [24].

For analysis, patients were stratified into colonic and rectal carcinoma, as these represent two distinct clinical entities with differing treatment modalities.

This study was approved by the Ethics Committee (3 July 2018).

### 2.1. Therapeutic Algorithm of Patients with Colorectal Carcinomas

All patients included in the study were treated according to the German S3 guidelines for colorectal cancer [3]. Consequently, colon carcinomas underwent primary surgery with adjuvant chemotherapy based on the postoperative histopathological findings. For rectal carcinomas staged as N+, T3/4, or located near the anal sphincter, neoadjuvant chemoradiation was administered prior to surgery. The decision to perform adjuvant therapy was determined based on the histopathological results.

### 2.2. Statistical Analysis

Statistical analyses were performed using SPSS Statistics (Version 28.0, IBM, Armonk, NY, USA). Continuous variables were tested for normality using the Shapiro–Wilk test. Variables following a normal distribution were analyzed using the independent samples *t*-test. Non-normally distributed variables were compared using the Mann–Whitney U test. The comparison of paired samples, such as quality of life data over time, was calculated using the Wilcoxon signed-rank test. The chi-square test was applied for categorical data. Statistical significance was set at *p* < 0.05.

## 3. Results

### 3.1. Patient Collective

A total of 99 patients were included in the study, all of whom met the inclusion criteria without fulfilling any exclusion criteria. The median patient age was 70 years, and 37% of the cohort were female. Among the participants, 56 (57%) had colonic carcinoma, while 43 (43%) had rectal carcinoma. Neoadjuvant treatment was administered to 24% of the patients, predominantly in the rectal carcinoma group (92%). The most common surgical procedure in the colonic carcinoma group was right hemicolectomy (70%), followed by left hemicolectomy (18%). In the rectal carcinoma group, the majority of patients underwent rectal resection (93%), while 7% required rectal extirpation. In the study cohort, 43% of patients underwent laparoscopic surgery, while 57% had an open approach. Postoperatively, 19% of all patients received adjuvant chemotherapy. A comprehensive summary of demographic, treatment, and histopathological characteristics is provided in Table 1.

### 3.2. Outcome Parameter After Colorectal Surgery for Colorectal Malignancy

#### 3.2.1. Colonic Carcinoma Group

Among the 56 patients with colonic carcinoma, 18 (32%) experienced postoperative morbidity with 7 (12%) classified as major morbidity (Clavien–Dindo grade III–V). Anastomotic insufficiency occurred in 5%, wound infection in 5%, and postoperative hematoma in 4% of patients. Reoperation was required in 9%, but no patient died postoperatively (0% mortality, see Table 2).

#### 3.2.2. Rectal Carcinoma Group

In the rectal carcinoma cohort, postoperative morbidity was reported in 14 (33%) patients with major morbidity affecting 5 (12%), and no postoperative deaths occurred (0% mortality). Anastomotic leakage, wound infection, and postoperative hematoma were each observed in 7% of patients. Additionally, 12% required reoperation (Table 2).

### 3.3. Quality of Life and Patient-Related Outcomes in the Entire Cohort

#### 3.3.1. Colonic Carcinoma Patients

Two functioning scales showed significant improvement from pretreatment to the one-year follow-up: global health status (63 vs. 72, *p* = 0.012) and emotional function (63 vs. 76, *p* = 0.009). Among symptom scales, five improvements (appetite loss, constipation, urinary frequency, abdominal pain, and blood/mucus in stool) and two deteriorations (anxiety and hair loss) were observed (Table 3).

#### 3.3.2. Rectal Carcinoma Patients

In contrast, rectal carcinoma patients experienced a decline in quality of life with two functioning scales worsening significantly: physical function (89 vs. 83, *p* < 0.001) and role function (81 vs. 68, *p* = 0.009). Symptom scales showed 12 deteriorations (pain, fatigue, financial impact, anxiety, buttock pain, trouble with taste, flatulence, fecal incontinence, sore skin around anus/stoma, stool frequency, embarrassed by defecation pattern/stoma, and impotence) but only two improvements (body image and blood/mucus in stool) (Table 3).

### 3.4. Quality of Life and Patient-Related Outcomes Stratified to Postoperative Morbidity

#### 3.4.1. Colonic Carcinoma Patients

Patients without postoperative morbidity demonstrated three improvements (appetite loss, abdominal pain, blood/mucus in stool) and three deteriorations (cognitive function, anxiety and hair loss) in quality of life items from pretreatment to the one-year follow-up. Patients with postoperative morbidity, however, showed four significant improvements, including global health status (59 vs. 77, *p* = 0.004), emotional function, constipation and urinary frequency with only one deterioration (anxiety).

When comparing all quality of life parameters, there were no significant differences between colonic carcinoma patients with and without morbidity either at pretreatment or the one-year follow-up (Table 4).

#### 3.4.2. Rectal Carcinoma Patients

In the group without morbidity, quality of life measures showed one improvement (blood/mucus in stool) and ten deteriorations (physical function, role function, fatigue, anxiety, body image, buttock pain, flatulence, fecal incontinence, sore skin around anus/stoma, and embarrassed by defecation pattern/stoma). Conversely, patients with morbidity experienced no improvements and two deteriorations (stool frequency and embarrassment due to defecation patterns/stoma).

Baseline pretreatment scores differed significantly between rectal carcinoma patients with and without morbidity in global health status, role function, fatigue, and sleep disturbance. At the one-year follow-up, dysuria, embarrassment by defecation pattern/stoma, and impotence were significantly more prevalent in the morbidity group (Table 5).

## 4. Discussion

In addition to survival, minimizing symptoms and functional impairments are essential goals in the treatment of colorectal carcinoma [3]. Postoperative complications often have significant short-term consequences for affected individuals, making them potential factors influencing mid- and long-term quality of life and function [17,18]. In this study, we investigated the impact of postoperative morbidity on mid-term quality of life and patient-related outcomes in a cohort of 99 patients undergoing surgery for colorectal carcinoma. Postoperative complications occurred in approximately one-third of carcinoma patients. Importantly, we observed a divergent pattern in quality of life outcomes between the two cancer subgroups. Patients with colonic carcinoma showed significant improvements in global health status and emotional function one year after surgery, regardless of postoperative morbidity. Conversely, patients with rectal carcinoma experienced a decline in several domains of quality of life, especially in those without postoperative morbidity. Surprisingly, patients with rectal cancer who experienced complications showed fewer deteriorations than those without. Overall, postoperative morbidity was not significantly associated with a worse quality of life at one-year follow-up, suggesting that mid-term patient-reported outcomes may be influenced by additional factors beyond postoperative complications alone.

Postoperative morbidity in our cohort occurred in 32% of colonic carcinoma patients and 33% of rectal carcinoma patients, mostly as a minor morbidity (63%). These values are in line with previously described morbidity rates in the literature [25,26,27,28].

In our analysis, global quality of life did not change between pretreatment and the one-year follow-up in rectal carcinoma patients and improved in colonic carcinoma patients. This confirms the results of several studies, which indicate that one year after treatment start, the quality of life remains constant or recovers and reach levels that are comparable with those of a healthy reference population as well as other cancer survivors or even better [4,5,6,29].

However, we found some changes in functioning and symptom scales: while patients with colonic carcinoma showed an overall improvement in more quality of life items, including an improved emotional function, those with rectal carcinoma experienced a clear predominance of deteriorated items, particularly concerning physical function and bowel function, including flatulence, fecal incontinence, stool frequency, and embarrassment due to defecation pattern/stoma. Postoperative changes in defecation are frequently described in the literature for patients receiving rectal resection, supporting our findings [6,10,30]. Despite these bowel function changes having the potential to influence quality of life, this was not evident in our analysis [6,10,13]. The improved emotional situation in colonic carcinoma patients in our cohort can be attributed to enduring the therapy and the primary removal of the tumor, although it comes with increased anxiety about tumor recurrence, which in turn can negatively affect global quality of life, but this was not the case in our cohort [31]. Additionally, symptom changes, such as worsening of buttock pain, hair loss, and trouble with taste, can be well explained as sequelae of radiation and/or chemotherapy. Conversely, there were improvements in symptoms, like appetite loss, abdominal pain, and blood and mucus in stool, all of which can be caused by a colorectal tumor and improved through tumor removal.

Previous studies have already identified different parameters with significant influences on quality of life and/or patient-related outcomes: age, gender, education, socio-economic status, worse baseline scores, cancer recurrence, presence of stoma, as well as the already mentioned changes in bowel, urinary, and sexual functions [6,7,10,13,14]. Comparing patients with and without postoperative morbidity, our results indicate that postoperative complications do not negatively impact mid-term quality of life. This aligns with the findings of Bosma et al., whose work demonstrated that postoperative complications have a significant impact on quality of life as well as the frequency of anxiety and depression in the short-term period of 6 weeks postoperative, but this effect fades after one year [19,20]. In contrast, another study by Orive et al., which also examined the impact of postoperative complications on health-related quality of life, found a persistent significant negative impact of these complications on quality of life at one year after surgery [21].

In our cohort, colonic carcinoma patients with postoperative morbidity even experienced an improvement in both global health status and emotional function, which was not observed in the group without morbidity. This effect could be attributed to the psychological resilience developed by enduring and overcoming postoperative complications.

These diverging results in the literature underline the complexity of assessing long-term quality of life outcomes and suggest that different study characteristics may lead to differing conclusions. Several factors may explain these inconsistencies. First, sample size and statistical power vary across studies, potentially influencing the ability to detect small but clinically meaningful differences. Second, patient populations differ with respect to age distribution, comorbidities, and frailty—all of which may affect recovery trajectories. For example, recent findings have highlighted the particularly unfavorable outcomes of very young colorectal cancer patients, who are often diagnosed in advanced stages and experience worse survival and progression-free survival rates, possibly due to delayed diagnosis and aggressive tumor biology [32]. Third, variations in surgical technique—such as anastomosis type—can also influence short-term recovery and complication rates, potentially affecting early postoperative quality of life [33,34]. Thus, nuanced technical aspects of surgical care may contribute to the differences observed across studies. Fourth, the severity and nature of complications differ between studies; some focus only on major complications (e.g., Clavien–Dindo grade III–V), while others include both major and minor events. Fifth, timing of quality of life assessments and the tools used to measure quality of life may influence outcomes. For example, short-term effects might be overestimated in early assessments, while later effects—especially psychological or functional limitations—may be underestimated if follow-up is insufficiently long or infrequent. In our study, the limited number of cases prevented us from performing stratified analyses by complication severity. We also assessed quality of life only at baseline and at one-year follow-up, which may have missed relevant short-term changes or longer-term effects beyond one year. Future research with larger, multicenter cohorts and extended follow-up intervals is warranted to clarify the duration and clinical relevance of postoperative complications on quality of life. In addition, subgroup analyses focusing on vulnerable populations—such as elderly or frail patients—could help identify those at higher risk for lasting quality of life impairments.

This study benefits from a prospective design with standardized assessment of quality of life using validated instruments (EORTC QLQ-C29 and QLQ-C30) both before treatment and at a one-year follow-up. By analyzing outcomes separately for patients with and without postoperative morbidity in both colon and rectal cancer cohorts, the study provides a nuanced understanding of the mid-term impact of postoperative complications on patient-reported outcomes.

However, our study has some limitations that need to be acknowledged. Firstly, the number of patients included in this study was limited, particularly in the subgroup analysis of patients with and without postoperative morbidity. Secondly, the single-center design may introduce biases. Thirdly, we include all patients with colorectal carcinomas receiving different colorectal resection procedures, which limits comparability [35,36]. Fourthly, quality of life data is always dependent on patient willingness to participate, which may lead to selection bias favoring those more inclined to take part. Fifthly, minor complications likely have a much smaller impact on well-being compared to major morbidity. Consequently, a stratification between minor and major complications would be desirable. However, this approach would reduce validity due to the small sample size (*n* = 12) for major morbidity.

## 5. Conclusions

Our data suggest that postoperative morbidity after colorectal resections for colorectal malignancies is not associated with a worse quality of life or patient-reported outcomes at the one-year follow-up. However, further studies with larger patient cohorts are needed to confirm these findings.

## Figures and Tables

**Table 1:** Patient characteristics in patient with and without postoperative morbidity after colorectal surgery for colon or rectal carcinoma.

Patient Characteristics	Colon Carcinoma			Rectal Carcinoma		
	Patients Without Morbidity (*n* = 38)	Patients with Morbidity (*n* = 18)	*p*	Patients Without Morbidity (*n* = 29)	Patients with Morbidity (*n* = 14)	*p*
**Age (years), median (IQR)**	72 (12)	72 (9)	0.766	62 (11)	70 (10)	0.110
**Gender, *n* (%)**			0.563			0.744
Female	17 (45)	6 (33)		10 (35)	4 (29)	
Male	21 (55)	12 (67)		19 (65)	10 (71)	
**BMI (kg/m^2^), median (IQR)**	28.1 (6.3)	28.6 (5.1)	0.307	28.0 (9.8)	26.1 (7.3)	0.568
**ASA classification, *n* (%)**			0.744			0.640
I	3 (8)	0 (0)		4 (14)	1 (7)	
II	24 (63)	11 (61)		17 (59)	7 (50)	
III	10 (26)	7 (39)		8 (28)	6 (43)	
IV	1 (3)	0 (0)		0 (0)	0 (0)	
**Neoadjuvant treatment, *n* (%)**	0 (0)	2 (11)	0.099	15 (52)	7 (50)	1.000
**Surgical procedure**			0.523			**0.029**
Right hemicolectomy	26 (68)	13 (72)				
Left hemicolectomy	8 (21)	2 (11)				
Sigmoid resection	3 (8)	1 (6)				
Colectomy	1 (3)	2 (11)				
Rectal resection	-	-		29 (100)	11 (79)	
Rectal extirpation	-	-		0 (0)	3 (21)	
**Adjuvant treatment, *n* (%)**	8 (21)	7 (39)	0.202	3 (10)	1 (7)	1.000
**(y)pT, n (%)**			1.000			0.758
0/1/2	12 (32)	5 (28)		14 (48)	6 (43)	
3/4	26 (68)	13 (72)		15 (52)	8 (57)	
**(y)pN, *n* (%)**			0.524			1.000
0	29 (76)	12 (67)		24 (83)	12 (86)	
1/2	9 (24)	6 (33)		5 (17)	2 (14)	

**Table 2:** Complication characteristics of patients after colorectal surgery.

Complication Characteristics	Patients with Surgery for Colonic Malignancy (*n* = 56)	Patients with Surgery for Rectal Malignancy (*n* = 43)
**Morbidity, *n* (%)**	18 (32)	14 (33)
**Clavien–Dindo, *n* (%)**		
I	1 (2)	1 (2)
II	10 (18)	8 (19)
III	6 (11)	5 (12)
IV	1 (2)	0 (0)
V	0 (0)	0 (0)
**Wound infection, *n* (%)**	3 (5)	3 (7)
**Hematoma, *n* (%)**	2 (4)	3 (7)
**Anastomostic leakage, *n* (%)**	2 (4)	3 (7)
**Re-surgery, *n* (%)**	5 (9)	5 (12)

**Table 3:** Pre- and postoperative quality of life (EQL C29 and C30) of patients with colorectal surgery for colorectal malignancy.

QoL Parameter	Patients with Surgery for Colonic Malignancy (*n* = 56)			Patients with Surgery for Rectal Malignancy (*n* = 43)		
	Pre-Treatment	One-Year Follow-Up	*p*	Pre-Treatment	One-Year Follow-Up	*p*
***Functioning scales***						
Global health status/QoL, mean (SD)	63 (27)	72 (22)	**0.012**	70 (16)	67 (20)	0.263
Physical function, mean (SD)	80 (21)	79 (24)	0.748	89 (17)	83 (17)	**<0.001**
Role function, mean (SD)	73 (35)	78 (29)	0.430	81 (27)	68 (33)	**0.009**
Emotional function, mean (SD)	63 (28)	76 (24)	**0.009**	62 (23)	67 (28)	0.518
Cognitive function, mean (SD)	86 (23)	78 (26)	0.163	90 (18)	84 (20)	0.112
Social function, mean (SD)	76 (31)	74 (29)	0.792	74 (25)	67 (32)	0.081
***Symptom scales***						
Dyspnoea, mean (SD)	20 (30)	20 (27)	0.863	14 (23)	19 (24)	0.361
Pain, mean (SD)	21 (26)	15 (27)	0.170	10 (18)	25 (31)	**0.003**
Fatigue, mean (SD)	38 (31)	35 (27)	0.387	26 (22)	34 (25)	**0.002**
Sleep disturbance, mean (SD)	32 (35)	29 (37)	0.589	28 (30)	31 (33)	0.624
Appetite loss, mean (SD)	18 (33)	9 (21)	**0.006**	13 (24)	8 (23)	0.469
Nausea and vomiting, mean (SD)	4 (14)	5 (16)	1.000	2 (6)	2 (5)	1.000
Constipation, mean (SD)	16 (26)	5 (16)	**0.003**	8 (19)	16 (27)	0.094
Diarrhoea, mean (SD)	19 (32)	26 (26)	0.482	26 (30)	28 (28)	0.703
Financial impact, mean (SD)	7 (16)	7 (19)	0.687	8 (18)	21 (36)	**0.020**
Anxiety, mean (SD)	37 (35)	64 (31)	**<0.001**	36 (30)	47 (36)	**0.013**
Weight, mean (SD)	76 (35)	71 (29)	0.773	81 (22)	76 (23)	0.057
Body image, mean (SD)	83 (21)	82 (24)	0.986	86 (18)	74 (26)	**0.005**
Sexual interests (only men), mean (SD)	45 (27)	40 (35)	1.000	43 (30)	57 (27)	0.619
Sexual interests (only women), mean (SD)	24 (32)	24 (33)	0.563	24 (32)	33 (32)	1.000
High urinary frequency, mean (SD)	44 (30)	35 (21)	**0.019**	36 (26)	37 (23)	0.307
Urinary incontinence, mean (SD)	10 (20)	12 (22)	0.827	4 (13)	2 (8)	1.000
Dysuria, mean (SD)	3 (15)	0 (0)	0.500	5 (14)	4 (12)	1.000
Abdominal pain, mean (SD)	22 (26)	10 (19)	**0.002**	11 (17)	13 (17)	1.000
Buttock pain, mean (SD)	4 (10)	6 (15)	0.766	10 (22)	30 (33)	**<0.001**
Bloated feeling, mean (SD)	22 (31)	17 (24)	0.491	16 (21)	26 (26)	0.263
Blood and mucus in stool, mean (SD)	9 (18)	1 (6)	**0.003**	31 (32)	5 (11)	**<0.001**
Dry mouth, mean (SD)	23 (34)	22 (28)	0.974	15 (20)	21 (25)	0.728
Hair loss, mean (SD)	4 (14)	16 (28)	**0.027**	2 (11)	7 (22)	1.000
Trouble with taste, mean (SD)	8 (21)	15 (28)	0.315	6 (16)	13 (27)	**0.031**
Flatulence, mean (SD)	24 (26)	37 (26)	0.227	20 (23)	43 (31)	**0.002**
Faecal incontinence/leakage, mean (SD)	7 (15)	11 (24)	0.437	6 (19)	28 (32)	**0.002**
Sore skin around anus/stoma, mean (SD)	11 (18)	17 (26)	0.108	8 (18)	40 (38)	**<0.001**
Stool frequency/bag change, mean (SD)	13 (21)	20 (26)	0.180	31 (29)	44 (30)	**0.014**
Embarrassed by defaecation pattern/stoma, mean (SD)	3 (10)	8 (19)	0.148	7 (20)	31 (36)	**<0.001**
Impotence (only men), mean (SD)	46 (41)	61 (38)	0.563	37 (34)	47 (37)	**0.021**
Dyspareunia (only women), mean (SD)	5 (17)	8 (19)	1.000	0 (0)	12 (31)	0.500

Red = worsening, green = improvement.

**Table 4:** Pre- and postoperative quality of life (EQL C29 and C30) of patients with colorectal surgery for colonic malignancy stratified in patients without and with postoperative complications.

QoL Parameter	Patients Without Postoperative Complications (*n* = 38)			Patients with Postoperative Complications (*n* = 18)			Comparison of Groups	
	Pre-Treatment	One-Year Follow-Up	*p*	Pre-Treatment	One-Year Follow-Up	*p*	Pre-Treatment	One-Year Follow-up
***Functioning scales***								
Global health status/QoL, mean (SD)	65 (27)	70 (23)	0.486	59 (26)	77 (18)	**0.004**	0.426	0.288
Physical function, mean (SD)	80 (22)	81 (23)	0.789	79 (19)	76 (28)	0.406	0.760	0.608
Role function, mean (SD)	73 (35)	77 (30)	0.955	72 (34)	79 (28)	0.250	0.768	0.857
Emotional function, mean (SD)	63 (29)	73 (24)	0.108	63 (26)	82 (21)	**0.031**	0.858	0.289
Cognitive function, mean (SD)	87 (23)	78 (29)	**0.019**	84 (23)	77 (19)	0.828	0.671	0.470
Social function, mean (SD)	73 (33)	73 (30)	0.770	82 (25)	79 (26)	1.000	0.380	0.670
***Symptom scales***								
Dyspnoea, mean (SD)	18 (27)	20 (27)	0.415	24 (36)	21 (31)	0.250	0.749	0.925
Pain, mean (SD)	23 (26)	15 (25)	0.054	17 (25)	17 (34)	1.000	0.360	0.699
Fatigue, mean (SD)	36 (31)	34 (28)	0.893	41 (31)	37 (27)	0.125	0.547	0.591
Sleep disturbance, mean (SD)	28 (33)	31 (38)	0.640	41 (39)	23 (35)	0.125	0.267	0.624
Appetite loss, mean (SD)	18 (31)	8 (21)	**0.031**	22 (36)	12 (22)	0.188	0.833	0.561
Nausea and vomiting, mean (SD)	4 (13)	6 (19)	0.500	5 (16)	2 (5)	0.750	0.762	0.576
Constipation, mean (SD)	15 (26)	4 (11)	0.125	19 (26)	6 (13)	**0.031**	0.649	1.000
Diarrhoea, mean (SD)	17 (33)	23 (23)	0.200	22 (30)	33 (33)	0.563	0.304	0.435
Financial impact, mean (SD)	9 (19)	10 (21)	0.539	2 (8)	0 (0)	1.000	0.205	0.179
Anxiety, mean (SD)	36 (33)	66 (32)	**<0.001**	39 (40)	61 (29)	**0.008**	0.933	0.563
Weight, mean (SD)	78 (34)	72 (26)	0.614	72 (37)	67 (38)	0.875	0.508	0.907
Body image, mean (SD)	82 (23)	82 (25)	0.890	85 (16)	81 (20)	0.781	0.910	0.618
Sexual interests (only men), mean (SD)	52 (27)	52 (35)	0.833	33 (25)	39 (39)	1.000	0.064	0.442
Sexual interests (only women), mean (SD)	24 (32)	26 (35)	0.375	22 (34)	11 (19)	1.000	0.882	0.691
High urinary frequency, mean (SD)	39 (26)	33 (21)	0.139	53 (37)	42 (23)	**0.047**	0.126	0.332
Urinary incontinence, mean (SD)	7 (18)	15 (24)	0.273	15 (23)	3 (10)	0.312	0.202	0.189
Dysuria, mean (SD)	2 (8)	0 (0)	0.500	6 (24)	0 (0)	1.000	0.695	1.000
Abdominal pain, mean (SD)	22 (25)	9 (17)	**0.033**	24 (30)	12 (22)	0.094	0.877	0.846
Buttock pain, mean (SD)	4 (10)	6 (16)	0.750	4 (11)	6 (13)	1.000	1.000	1.000
Bloated feeling, mean (SD)	20 (30)	15 (24)	0.625	28 (33)	23 (27)	0.719	0.310	0.335
Blood and mucus in stool, mean (SD)	8 (16)	1 (3)	**0.008**	10 (21)	3 (10)	0.188	0.953	0.805
Dry mouth, mean (SD)	18 (30)	20 (28)	0.375	33 (40)	27 (29)	0.281	0.196	0.403
Hair loss, mean (SD)	4 (13)	13 (25)	**0.047**	6 (17)	24 (37)	0.500	0.897	0.315
Trouble with taste, mean (SD)	8 (23)	13 (29)	0.438	9 (19)	21 (27)	0.625	0.618	0.218
Flatulence, mean (SD)	25 (26)	32 (22)	0.895	22 (28)	52 (31)	0.063	0.589	0.071
Faecal incontinence/leakage, mean (SD)	8 (17)	10 (23)	0.883	6 (13)	15 (27)	0.500	0.761	0.514
Sore skin around anus/stoma, mean (SD)	11 (18)	16 (23)	0.157	9 (19)	21 (34)	0.531	0.558	0.816
Stool frequency/bag change, mean (SD)	11 (18)	21 (28)	0.171	15 (27)	18 (20)	0.781	0.890	0.978
Embarrassed by defaecation pattern/stoma, mean (SD)	2 (8)	5 (12)	0.375	6 (13)	15 (31)	0.500	0.317	0.478
Impotence (only men), mean (SD)	46 (40)	60 (35)	0.250	47 (44)	61 (49)	1.000	1.000	0.852
Dyspareunia (only women), mean (SD)	7 (19)	8 (20)	1.000	0 (0)	11 (19)	1.000	1.000	1.000

Red = worsening, green = improvement.

**Table 5:** Pre- and postoperative quality of life (EQL C29 and C30) of patients with colorectal surgery for rectal malignancy stratified in patients without and with postoperative complications.

QoL Parameter	Patients Without Postoperative Complications (*n* = 29)			Patients with Postoperative Complications (*n* = 14)			Comparison of Groups	
	Pre-Treatment	One-Year Follow-Up	*p*	Pre-Treatment	One-Year Follow-Up	*p*	Pre-Treatment	One-Year Follow-Up
***Functioning scales***								
Global health status/QoL, mean (SD)	73 (18)	69 (22)	0.208	64 (10)	63 (15)	1.000	**0.031**	0.213
Physical function, mean (SD)	93 (10)	85 (16)	**0.001**	80 (25)	78 (20)	0.148	0.054	0.332
Role function, mean (SD)	87 (24)	71 (31)	**0.009**	68 (30)	58 (38)	0.375	**0.010**	0.392
Emotional function, mean (SD)	66 (19)	70 (28)	0.709	55 (30)	58 (27)	0.750	0.179	0.218
Cognitive function, mean (SD)	90 (19)	84 (23)	0.146	89 (18)	83 (13)	0.875	0.711	0.561
Social function, mean (SD)	79 (22)	71 (31)	0.191	65 (29)	54 (33)	0.240	0.146	0.126
***Symptom scales***								
Dyspnoea, mean (SD)	12 (18)	20 (24)	0.332	19 (31)	17 (25)	1.000	0.622	0.892
Pain, mean (SD)	8 (15)	19 (26)	0.066	15 (22)	42 (37)	0.063	0.224	0.086
Fatigue, mean (SD)	19 (16)	31 (23)	**0.006**	39 (27)	42 (28)	0.250	**0.020**	0.368
Sleep disturbance, mean (SD)	20 (26)	26 (29)	0.756	43 (33)	46 (40)	1.000	**0.027**	0.210
Appetite loss, mean (SD)	7 (17)	5 (16)	0.750	24 (33)	17 (36)	0.750	0.070	0.432
Nausea and vomiting, mean (SD)	2 (7)	2 (5)	1.000	1 (4)	2 (6)	1.000	1.000	1.000
Constipation, mean (SD)	8 (19)	11 (22)	0.375	10 (20)	29 (38)	0.125	0.947	0.119
Diarrhoea, mean (SD)	26 (30)	30 (27)	0.578	24 (30)	21 (31)	1.000	0.759	0.336
Financial impact, mean (SD)	8 (17)	17 (30)	0.125	7 (19)	33 (47)	0.250	0.723	0.419
Anxiety, mean (SD)	37 (27)	53 (34)	**0.004**	36 (36)	29 (38)	1.000	0.710	0.112
Weight, mean (SD)	82 (21)	80 (22)	0.398	81 (25)	63 (21)	0.125	1.000	0.067
Body image, mean (SD)	88 (17)	79 (20)	**0.044**	83 (19)	60 (34)	0.078	0.451	0.153
Sexual interests (only men), mean (SD)	52 (26)	57 (20)	0.922	26 (32)	56 (40)	0.250	0.060	1.000
Sexual interests (only women), mean (SD)	27 (33)	30 (31)	1.000	17 (33)	44 (38)	1.000	0.868	0.773
High urinary frequency, mean (SD)	32 (26)	34 (24)	0.624	44 (26)	44 (20)	0.500	0.153	0.297
Urinary incontinence, mean (SD)	2 (9)	2 (7)	1.000	7 (19)	4 (12)	1.000	0.436	1.000
Dysuria, mean (SD)	2 (9)	2 (7)	1.000	10 (20)	12 (17)	0.500	0.248	**0.048**
Abdominal pain, mean (SD)	11 (18)	12 (16)	1.000	10 (16)	17 (18)	1.000	0.916	0.678
Buttock pain, mean (SD)	7 (16)	29 (35)	**0.004**	17 (31)	33 (31)	0.125	0.291	0.649
Bloated feeling, mean (SD)	15 (21)	23 (26)	0.425	17 (22)	33 (25)	0.625	0.893	0.264
Blood and mucus in stool, mean (SD)	33 (32)	5 (12)	**0.003**	27 (32)	4 (8)	0.063	0.623	1.000
Dry mouth, mean (SD)	13 (21)	21 (24)	0.848	19 (17)	21 (31)	0.625	0.181	0.833
Hair loss, mean (SD)	0 (0)	5 (21)	1.000	7 (19)	13 (25)	1.000	0.106	0.166
Trouble with taste, mean (SD)	5 (15)	12 (24)	0.125	7 (19)	17 (36)	0.500	0.895	1.000
Flatulence, mean (SD)	23 (25)	43 (32)	**0.027**	14 (17)	42 (30)	0.094	0.348	1.000
Faecal incontinence/leakage, mean (SD)	9 (22)	30 (35)	**0.016**	0 (0)	21 (25)	0.125	0.158	0.666
Sore skin around anus/stoma, mean (SD)	6 (16)	41 (36)	**<0.001**	12 (21)	38 (45)	0.188	0.369	0.693
Stool frequency/bag change, mean (SD)	32 (31)	44 (29)	0.114	27 (25)	46 (34)	**0.031**	0.870	0.877
Embarrassed by defaecation pattern/stoma, mean (SD)	7 (21)	24 (34)	**0.028**	7 (19)	50 (36)	**0.016**	0.858	**0.048**
Impotence (only men), mean (SD)	33 (30)	36 (31)	0.109	44 (41)	72 (39)	0.250	0.569	**0.027**
Dyspareunia (only women), mean (SD)	0 (0)	17 (36)	0.500	0 (0)	0 (0)	1.000	1.000	0.782

Red = worsening, green = improvement, blue = significant difference.

## Data Availability

All data have been included in the manuscript and the tables.
